# Transportation of Nanoscale Cargoes by Myosin Propelled Actin Filaments

**DOI:** 10.1371/journal.pone.0055931

**Published:** 2013-02-21

**Authors:** Malin Persson, Maria Gullberg, Conny Tolf, A. Michael Lindberg, Alf Månsson, Armagan Kocer

**Affiliations:** 1 School of Natural Sciences, Linnaeus University, Kalmar, Sweden; 2 Biochemistry Department, University of Groningen, Groningen, The Netherlands; Dalhousie University, Canada

## Abstract

Myosin II propelled actin filaments move ten times faster than kinesin driven microtubules and are thus attractive candidates as cargo-transporting shuttles in motor driven lab-on-a-chip devices. In addition, actomyosin-based transportation of nanoparticles is useful in various fundamental studies. However, it is poorly understood how actomyosin function is affected by different number of nanoscale cargoes, by cargo size, and by the mode of cargo-attachment to the actin filament. This is studied here using biotin/fluorophores, streptavidin, streptavidin-coated quantum dots, and liposomes as model cargoes attached to monomers along the actin filaments (“side-attached”) or to the trailing filament end via the plus end capping protein CapZ. Long-distance transportation (>100 µm) could be seen for all cargoes independently of attachment mode but the fraction of motile filaments decreased with increasing number of side-attached cargoes, a reduction that occurred within a range of 10–50 streptavidin molecules, 1–10 quantum dots or with just 1 liposome. However, as observed by monitoring these motile filaments with the attached cargo, the velocity was little affected. This also applied for end-attached cargoes where the attachment was mediated by CapZ. The results with side-attached cargoes argue against certain models for chemomechanical energy transduction in actomyosin and give important insights of relevance for effective exploitation of actomyosin-based cargo-transportation in molecular diagnostics and other nanotechnological applications. The attachment of quantum dots via CapZ, without appreciable modulation of actomyosin function, is useful in fundamental studies as exemplified here by tracking with nanometer accuracy.

## Introduction

Intracellular transport of nanoscale cargoes (lipid vesicles, mitochondria *etc.*) is executed by molecular motors [1], [2] such as kinesins and dyneins, walking along tracks of microtubules and myosins walking along actin filaments. This has inspired development of molecular motor driven lab-on-a-chip devices [3], [4], [5], [6], [7] with cargo pick-up and transportation [8], [9], [10], [11], [12], [13], [14], [15], [16], [17]. Possible applications include periodic chemistry [18], [19], assembly of molecular components [20], [21], [22], sorting, positioning and/or concentration [20], [23], [24], [25], [26], [27] of molecules. Even proof-of-principle diagnostics devices [28], [29], [30], [31], [32] have been described, with significant future potential [3], [7]. In the mentioned applications, cargo-transportation usually relies on surface immobilization of the motors with microtubules and actin filaments as “shuttles” with attached cargoes of nanometer (e.g. oligonucleotides, proteins or nanoparticles) to micrometer size (e.g. polystyrene beads, bacteria or large liposomes) [10], [12], [13], [14], [21], [22], [29], [33], [34], [35], [36], [37], [38], [39], [40], [41]. In a vast majority of these studies the microtubule-kinesin motor system has been employed, partly due to the belief that effective cargo-transportation is considerably more challenging with actomyosin [7], [11], [42], [43], e.g. related to possible rotation of the filaments around their long axis [44], [45]. Such rotation may be deleterious if bulky cargoes are attached to the actin monomers along the filament length (“side-attached”). Accordingly, in several recent studies the cargoes have been attached either to bundled actin filaments [15], [37] (with increased complexity) or to the trailing end (“end-attached) of the filament via the capping protein gelsolin [10], [33], [40]. Even though the latter approach would alleviate rotation-induced clashes with a dense motor layer, the small capacity for cargo loading would be a limitation in nanotechnological applications. On the other hand, the attachment to the trailing end may be useful for fundamental studies, e.g. labelling of the filament plus end [46], tracking with nanometer accuracy [47], [48] and application of loads to the actin filaments via magnetic particles [10]. However, in this case it is important to ensure that the capping protein itself does not affect the actomyosin function, a possibility with gelsolin [49] since actin filament structure is modified by interactions of gelsolin with the actin monomer at the filament end [50], [51], [52], [53].

In view of the ten-fold higher speed of actomyosin driven transportation compared to microtubule-kinesin driven transportation and the perceived [7] difficulties in using actin filaments as shuttles, it is of interest to define the maximum capacity of cargo transportation with side-attached cargoes of different size for effective use in various nanodevices. Based on limited evidence in the literature [11], [32], [37], we hypothesize that the capacity is high for protein-sized cargoes, but quite small for slightly larger cargoes (e.g. quantum dots), due to rotation of the filament around its long axis during sliding. We also hypothesize that any steric clashes due to rotation would be eliminated if the nanoparticles were attached to the trailing end of actin filaments via a suitable capping protein. Particularly, we postulate that CapZ, not previously tested for this purpose, would be suitable. Thus, unlike the capping protein gelsolin, CapZ does not sever actin filaments suggesting that structural changes in the filaments are more limited.

In order to address the above hypotheses, we here study actomyosin-based transportation of side-attached and end-attached model cargoes, including streptavidin, quantum dots and liposomes, of sizes comparable to diagnostically relevant antibodies, viruses and microvesicles, respectively (Fig. S1). Furthermore, we show, for the first time, transportation of cargoes attached to the trailing end of actin filament via the plus end actin capping protein CapZ [54], [55], [56]. The results with side-attached cargoes show that >1000 covalently immobilized biotins, >50 streptavidin molecules, >4 quantum dots and liposomes may be transported at similar velocity as in the absence of cargo. However, the fraction of motile filaments was reduced with increased degree of cargo loading. Whereas only limited effect was seen for up to 50 streptavidin molecules, quite severe effects were observed for just a few quantum dots or single liposomes. The results with end-attached cargoes suggest that CapZ does not cause noticeable effect on motility. The results are discussed in relation to future development of actomyosin-powered nanodevices and the use of nanoparticle-attachment in fundamental studies. Key results that shed new light on important mechanisms of actomyosin based motion generation are also discussed.

## Materials and Methods

### Ethics Statement

All experiments using animal material were approved by the Regional Ethical Committee for Animal experiments in Linköping, Sweden (reference numbers: 52–05, 58–08 and 96–11) and performed in accordance with national and EU legislations.

### Abbreviations

A list of abbreviations (Abbreviations S1) is given in the Supporting Information.

### Solutions and Chemicals

Actin monomers (G-actin) were dissolved in G-buffer: 2 mM tris-HCl pH 7.6, 0.2 mM adenosine-5′-triphosphate (ATP), 0.2 mM CaCl_2_, 0.2 mM Ditiothreitol (DTT) and 0.2 mM NaN_3_. Heavy meromyosin (HMM) and other proteins were diluted in a buffer A: 10 mM 3-morpholinopropane-1-sulfonic acid (pH 7.4), 1 mM MgCl_2_, 0.1 mM ethylene glycol tetraacetic acid, 1–10 mM DTT and appropriate amounts of KCl to produce ionic strengths in the range 40–130 mM. All chemicals were of analytical grade and, if not otherwise stated, purchased from Merck (Darmstadt, Germany), Sigma-Aldrich (St. Louis, MO, USA) or Fluka and Riedel-de Haën (Seelze, Germany). Alexa Fluor® 488 phalloidin (APh), rhodamine phalloidin (RhPh) and Qdot® 605 Streptavidin conjugate (quantum dot) were all purchased from Invitrogen - Molecular Probes (Eugene, OR, USA) while tetramethylrhodamine-isothiocyanate (TRITC)-streptavidin was purchased from Pierce (Rockford, IL, USA).

### Protein Preparations

Actin and myosin II were isolated from rabbit back muscle and rabbit fast leg muscle [57]. Myosin was digested by α-chymotrypsin to prepare HMM [58], whereas actin was purified essentially as in [59] with special care taken to remove tropomyosin, troponins and other soluble proteins. The G-actin was stored on ice and used within a week. Alternatively, G-actin or actin filaments (F-actin) were frozen in liquid nitrogen and stored at −80°C until use (cf. [60]).

CapZ was expressed in *Escherichia coli* (*E. coli*) and purified as described previously [54]. Briefly, the expression was induced with 0.1 mM isopropyl β-D-thiogalactopyranoside for 3 h before lysis of cells and purification on a HisTrap™ HP column (GE Healthcare Bio-Science AB, Uppsala, Sweden) according to manufactureŕs protocol. The purity of the actin and CapZ preparations are illustrated by the sodium dodecyl sulphate polyacrylamide gel electrophoresis (SDS-PAGE) in Fig. S2.

### Protein Biotinylation and Covalent Rhodamine Labelling

Actin and CapZ were biotinylated with *N*-Hydroxysulfosuccinimide (NHS) ester coupling chemistry using an EZ-Link Sulfo-NHS-LC-Biotinylation kit (Pierce) according to manufactureŕs protocol (see also [61]). Alternatively, CapZ was labelled with rhodamine using a NHS-Rhodamine antibody labelling kit (Pierce) according to manufacturer’s protocol resulting in 1–2 rhodamines per CapZ molecule. The extent of biotinylation according to 4′-hydroxyazobenzene-2-carboxylic acid assay was 0.8–1 biotins per actin monomer (unless otherwise stated) and 4–6 biotins per CapZ molecule. In the experiments using fluorescent TRITC-streptavidin there were 4 biotins per actin monomer and 2 TRITC molecules, on average, per streptavidin.

### Biotinylated Liposome Preparation

All lipids were purchased from Avanti Polar Lipids Inc. Alabaster, Alabama. Liposomes were prepared from 1,2-Diphytanoyl-sn-Glycero-3-Phosphocholine, 1-Palmitoyl-2-Oleoyl-sn-Glycero-3-[Phospho-rac-(1-glycerol)], cholesterol and 1,2-distearoyl-*sn*-glycero-3-phosphoethanolamine-N-[biotinyl(polyethylene glycol)-2000] (ammonium salt) with a ratio 70∶25:5∶1 wt. % as explained before [62]. Briefly, lipids from chloroform stocks were mixed at the desired weight ratio and a thin lipid film was obtained by evaporating the chloroform in a rotary evaporator. The lipid film was rehydrated at 50°C for 45 min by adding 1 ml buffer (10 mM Tris, pH 7.5). After 5 freeze and thaw cycles in liquid nitrogen and 50°C water-bath, respectively, the liposomes were stored at −80°C for further use. Before experiments, pre-warmed liposomes (10 mg/ml) were extruded 11 times through a 400 nm pore size polycarbonate filter (Avestin) and the resulting large unilamellar vesicles were titrated at 50°C with Anapoe®-X-100 (Triton X-100) until saturation. The detergent-destabilized liposomes were mixed with 1 volume of 200 mM calcein (Na-salt) (Sigma). Detergent removal was achieved by incubating the mixture with 200 mg wet weight of Bio-Beads SM-2 adsorbents (Bio-Rad Laboratories, Hercules, CA, USA) at 4°C. This procedure yields biotinylated unilamellar liposomes (average diameter of 100 nm) with 100 mM fluorescent calcein inside [63].

### *In vitro* Motility Assay – General Aspects

All dilutions of proteins and washing steps were performed using buffer A with ionic strength of 60–80 mM unless otherwise stated. *In vitro* motility assays were performed according to standard methods [42], [58], [61], where 50–120 µg/ml rabbit HMM was first adsorbed to cover glasses derivatized with trimethylchlorosilane (TMCS, Sigma; [43], [64]). Subsequently, the *in vitro* motility assay flow cell was incubated with 1 mg/ml biotin-free bovine serum albumin fraction V (BSA; Sigma-Aldrich) for 30 s – 1 min. In some experiments, ATP insensitive “rigor heads” of HMM were either removed by affinity precipitation using ultracentrifugation in the presence of F-actin and ATP [65] or by incubating 1 µM non-fluorescent “blocking actin” directly in the flow cell before introducing fluorescent/biotinylated F-actin (2–10 nM; monomer concentration). F-actin was labelled with RhPh or APh as in [58]. All assay solutions contained 1 mM MgATP (unless otherwise stated), had pH 7.4 and were supplemented with an oxygen scavenger system (3 mg/ml glucose, 100 µg/ml glucose oxidase, 20 µg/ml catalase, 10 mM DTT). The ionic strength was either 40 (A40), 60 (A60), 80 (A80), 90 (AMc90) or 130 mM (AMc 130) as adjusted with KCl. In the AMc90 and AMc130 assay solutions, 0.5–0.6% methylcellulose (M0262, Sigma-Aldrich; 2% aqueous solution gives viscosity 400cP) was included to prevent filaments from diffusing away from the surface. The motility assays were generally performed at room temperature (21–23°C) but the temperature was in some experiments, increased to 25 or 29°C. The range of temperatures used, gave sliding velocities in the range of 2 to 10 µm/s. Importantly, the temperature was constant within ±0.5°C during each given experiment.

Samples were observed using an inverted epifluorescence microscope (Eclipse TE 300, Nikon, Tokyo, Japan) equipped with an oil immersion objective (100x, NA 1.40, Nikon). Alternatively, an objective based total internal reflection fluorescence (TIRF) microscope was used. This was built around the microscope using a TIRF objective (60x, NA 1.49, Nikon) and a 532 nm diode laser for illumination. Actin filaments that were labelled with RhPh, TRITC-streptavidin or quantum dot were visualized using a TRITC filter-set (Ex. 540/25, DM 565, BA 605/55). APh-labelled actin filaments or calcein loaded liposomes were visualised by a fluorescein isothiocyanate (FITC) filter-set (Ex. 465–495, DM 505, BA 515–555). Images were acquired using an electron multiplying charge coupled device (EMCCD) camera (C9100-12, Hamamatsu Corporation, Hamamatsu, Japan). The sequence of images was captured at ∼5 s^−1^. The sliding velocities were estimated from these image sequences using a manual version of a previously described tracking program [66], [67]. In this software, an algorithm searches for the 10 frames in a row, where the coefficient of variation (CV) of the frame-to-frame velocity is lowest (cf. Fig. S3). If not otherwise stated, the velocity data below refer to the mean value for several filaments of such 10-frame averages with CV <0.5. Another measure of velocity, the average velocity (Fig. S3A), was obtained by dividing the distance moved with the time (generally in the range of 5–20 s) over which the movement took place. Here, all moving (but not entirely stationary) filaments were included in the analysis whether they moved smoothly or not. When different conditions (*e.g*. different number of quantum dots per filament) were compared, similar time (number of image frames) was used for all conditions. For velocity-length plots, actin filament lengths were obtained from filament intensity as described previously [42].

### *In vitro* Motility Assay with Nanoscale Cargoes Attached along Actin Filaments

TRITC-streptavidin (1–100 nM) or streptavidin coated quantum dots (2–10 nM) were incubated for 1 min in flow cells with biotinylated actin filaments attached to HMM. The flow cell was washed before the motility was initiated by addition of the different assay solutions (A40, A80, or AMc130). For experiments in which free biotin in solution was used to block the unoccupied biotin-binding sites of streptavidin, the flow cell was washed with 20 nM–40 µM biotin before the motility was initiated by addition of AMc130. This gave at least 8 times higher concentration of free biotin than the incubated streptavidin concentration.

For liposome attachment, RhPh labelled and biotinylated F-actin was first immobilised on HMM before incubation with non-fluorescent streptavidin (24 nM) for 1 min. Subsequently, two rinsing steps were imposed before calcein filled liposomes were added to the flow cell. Finally, the flow cell was further washed two times before the motility was initiated by infusing AMc130 solution.

### *In vitro* Motility Assay with Nanoscale Cargoes Attached to the Trailing End of Actin Filaments

Actin filaments capped with CapZ were obtained by having either biotinylated or rhodamine labelled CapZ present during polymerization of G-actin to F-actin for 3 h. The polymerization was initiated by addition of KCl, MgCl_2_ and ATP to the final concentrations 100 mM, 2 mM and 3.3 mM, respectively, in G-actin buffer and APh was added at equimolar concentrations to achieve fluorescence labelling. The filaments were used the same day in the *in vitro* motility assay. When using filaments with biotinylated CapZ, quantum dots (1–10 nM) were incubated in the flow cell for 30 s – 1 min. Subsequently, the flow cell was washed before the motility was initiated by addition of the different assay solutions (A60, AMc90 or AMc130). For the nanometer tracking experiments, AMc130 solution was used with MgATP concentrations ranging from 0 to 50 µM.

### Number of Fluorescent Streptavidin Molecules Per µm of an Actin Filament

The number of TRITC-streptavidin molecules along an actin filament was calculated from the background-subtracted fluorescence intensity on the assumption of 2 TRITC-molecules per streptavidin (according to manufactureŕs specification). The intensity attributed to each TRITC-molecule was obtained from the fluorescence intensity per RhPh in RhPh labelled F-actin with the assumptions of 1.360 RhPh molecules/ µm and 2. the same fluorescence intensity per RhPh as per TRITC molecule on streptavidin. The validity of these assumptions is supported by a recent analysis [32].

### Tracking with nm Accuracy

Quantum dots attached to the trailing end of actin filaments via CapZ were tracked by fitting a two-dimensional Gaussian function [47] to the diffraction limited intensity distribution of the quantum dot. The image frames in these experiments were captured using an EMCCD chip (C9100-12, Hamamatsu; see above) with a pixel size corresponding to 165×165 nm^2^ on the flow cell using a 100×objective (NA 1.4). The frame rate was 5 frames/s. Repeated measurements using stationary quantum dots suggested a tracking accuracy of ±5 nm (standard deviation).

### Data Analysis

Data are expressed as mean ± standard error of the mean (SEM) or as mean ±95% confidence interval (CI) as indicated. Linear and non-linear regression and other statistical analyses were performed using GraphPad Prism software (version 5.01, GraphPad Software, San Diego, CA, USA).

## Results

### Negligible Effects on Motility of more than Thousand Small Molecules and Tens of Streptavidin Molecules Per µm of an Actin Filament

**Figure 1:**
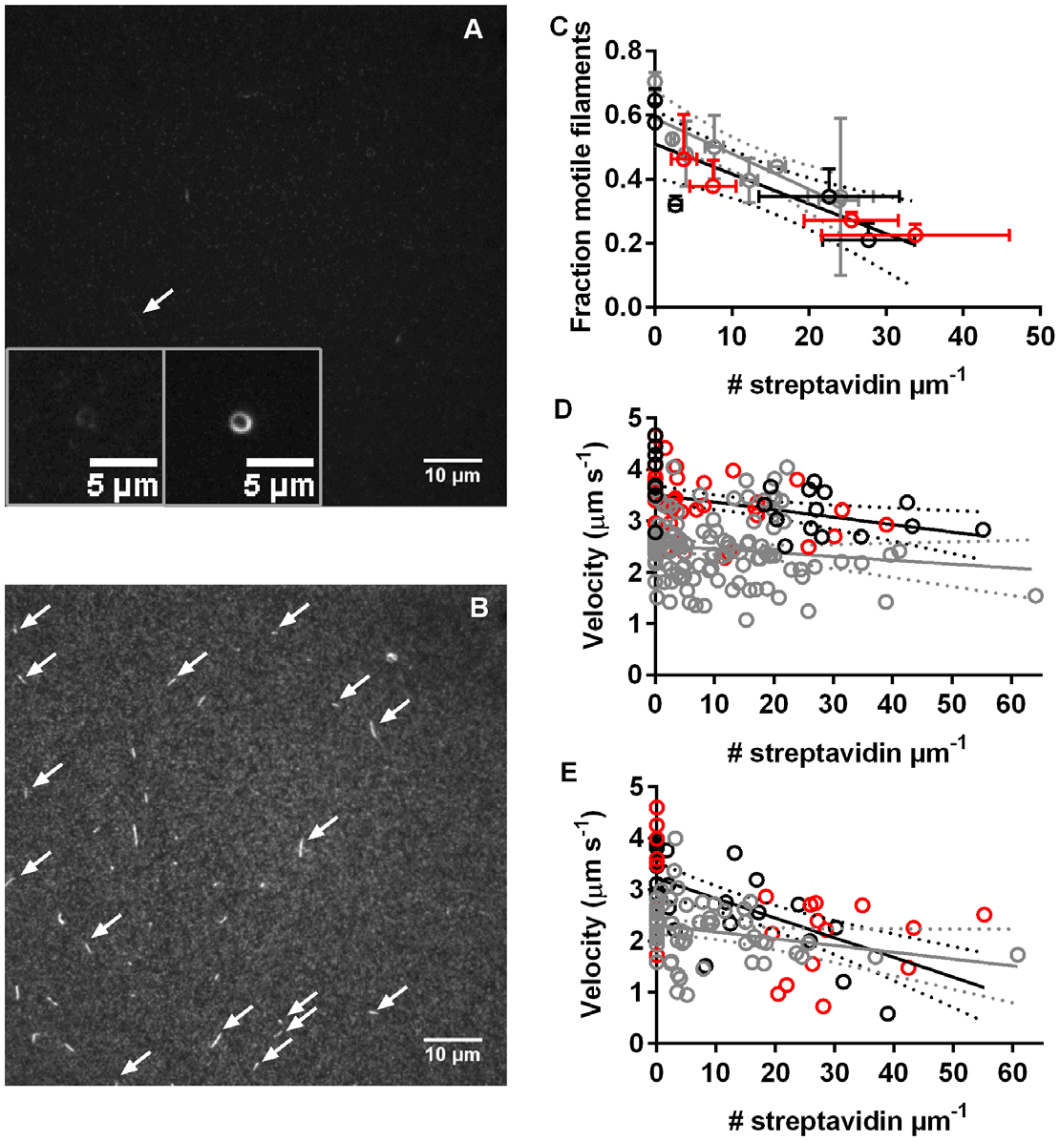
Effect of different degrees of streptavidin labelling on HMM induced actin filament motility. **A.** Biotinylated and APh labelled F-actin (5 nM), immobilized to HMM on a surface, then incubated with 1 nM TRITC-streptavidin for 1 min followed by rinsing and incubation by AMc130 assay solution. Observation using TRITC filter set. Arrow indicates motile TRITC-streptavidin labelled filament. Other filament-like objects represent cross-linked and non-motile filaments. *Inset*: nanospool of actin filament formed by intra-filament biotin-streptavidin cross-linking. Left image, TRITC-filter set to observe TRITC-streptavidin. Right image, FITC-filter set to observe Alexa-488-labelling of the same filament. **B.** Same conditions as in A except for incubation with 20 nM TRITC-streptavidin and 40 µM biotin in solution. Arrows indicate motile filaments. Note, brighter background than in A, presumably due to non-specific binding of TRITC-streptavidin. **C.** Fraction of motile filaments vs. average number of streptavidin molecules per µm filament length. Vertical error bars: SEM. Horizontal error bars: standard deviation. **D.** Velocity for filaments with different number of streptavidin molecules measured during the periods of smoothest sliding (CV <0.5). **E.** Average velocity during 6–16 s plotted against number of streptavidin molecules per filament length. Black and grey symbols: two different experimental dates with different HMM, actin and streptavidin batches. Red symbols: covalently biotinylated F-actin with addition of 0–20 nM TRITC-streptavidin followed by 20 nM to 40 µM biotin, prior to motility assay. Same experimental date as black. Straight full lines obtained by linear regression as well as dashed lines representing 95% confidence intervals.

The approximately 360 actin monomers per µm of an actin filament [68], [69], each with reactive lysines and cysteines, serve as regularly spaced and naturally occurring sites for covalent modification [32], e.g. biotinylation. Thus, in order to attach streptavidin-labelled cargoes to F-actin, the filaments were first covalently biotinylated with 1–4 biotins per actin monomer (∼360–1500 per µm of the filament). In these and other experiments in the present study, the biotins were attached to lysines on actin and both biotinylation and subsequently streptavidin binding would be most likely for the most accessible lysines in the order: (i). Lys-113, (ii) Lys 215 and (iii) Lys-50, Lys-191, Lys-284, Lys-291, Lys-315, Lys-326 and Lys-359 (Analysis using Swiss-Pdb Viewer 4.0.44 http://www.exapzy. org/spdbv/ of Protein data bank entry 1M8Q). This biotinylation (Fig. 1, Table 1) had, in itself, negligible effects on the HMM induced actin sliding velocity and on the fraction of motile filaments whether pre-incubation with blocking actin (to block rigor-like heads) was employed or not. Moreover (Table 1), actin filaments exhibited similar sliding velocities (cf. [60]) and fractions of motile filaments whether they were labelled with APh or RhPh. To study the interaction between biotinylated actin filaments and fluorescent streptavidin (∼5 nm in diameter), biotinylated actin filaments (4 biotins per actin) were first immobilized to HMM on the surface before incubation with 1–100 nM TRITC-streptavidin. This caused appreciable concentration dependent (Fig. 1A, B) streptavidin labelling (5–60 streptavidin molecules/ µm). In addition, there was non-specific binding of TRITC-streptavidin to the HMM coated surface (Fig. 1 A–B) even if BSA had been used to block non-specific binding. In the absence of free biotin in the solution (e.g. Fig. 1A; Movie S1) there was increased tendency for blocking of the motility by inter- and intrafilament cross-linking via biotin-streptavidin-biotin links. This was particularly seen at low streptavidin loading of the filaments with a large number of free biotin molecules accessible on actin. An observation worth noting in this connection, is the self-assembly of actin filament spools (insets Fig. 1A, Movie S1; see previous microtubule results [70]) stabilized by intrafilament biotin-streptavidin mediated cross-links particularly seen with low streptavidin loading on actin. Addition of biotin to the streptavidin labelled filaments under these conditions reduced the actin filament cross-linking via streptavidin-biotin links (Fig. 1A, C). A similar effect of biotin was not observed at high streptavidin loading (Fig. 1B, C; Movie S2).

**Table 1:** Effect of covalent biotinylation and quantum dot attachment on HMM induced actin sliding.

	Velocity (mean ± SEM; µm/s)^1^/fraction of motile filaments	
Sample	With blocking actin	Without blocking actin
Actin- RhPh	2.02±0.09 (n = 20)/80% motile	2.06±0.04 (33)/71% motile
Actin-APh	2.01±0.1 (24)/73% motile	2.14±0.07 (23)/75% motile
Biotin-actin-RhPh	1.97±0.07 (96)/83% motile	1.93±0.04 (37)/75% motile
Biotin-actin-APh	1.90±0.07 (51)/77% motile	1.77±0.1 (26)/77% motile
Biotin-actin-APh +1 quantum dot	1.95±0.09 (17)/41% motile	1.84±0.08 (52)/66% motile

1 A80 solution. No methylcellulose in assay solution. Motility assay performed at room temperature (21–23°C) and kept constant within ±0.5°C during each experiment. Number of filaments used for velocity measurements in parentheses. RhPh: rhodamin-phalloidin; APh: AlexaFluor® 488 phalloidin.

The sliding velocity during periods of smooth sliding (CV of frame-to-frame velocity <0.5) was only negligibly affected by binding of 1–60 streptavidins/ µm (Fig. 1D). In contrast, the average velocity (including periods with temporarily halted motility) was reduced with increased number of streptavidin molecules in one of the two separate experiments (Fig. 1E). This is consistent with the increased fraction of stationary filaments, which, however, was observed in both experiments (Fig. 1C).

### Side-attached Quantum Dots have Minimal Effects on Sliding Velocity

Next, we studied the effect of larger cargoes by attachment of streptavidin coated semiconductor quantum dots to the filaments. The quantum dots had 5–10 streptavidin molecules immobilized on the surface and exhibited smaller and large axes diameters of 15 and 20 nm, respectively (manufactureŕs specifications). The approximate diameters in the absence of streptavidin were 5 and 12 nm, respectively. In a first set of experiments, biotinylated actin filaments were incubated for ∼2 min with streptavidin coated quantum dots (2 nM) while immobilised to HMM in the *in vitro* motility assay flow cell. This led to binding of mostly one, but occasionally two quantum dots to between 3 and 15% of the filaments in 6 different experiments (mean 7.5±1.7%; Movie S3). A similar total number of non-specifically bound quantum dots were observed on the flow cell surface outside the biotinylated filaments. The sliding velocity was not appreciably affected by the quantum dot attachment whether pre-incubation with blocking actin (non-fluorescent actin filaments to block dead heads) was employed or not (Table 1, last row). Moreover, in great contrast with previous studies using quantum dot-attachment to actin filaments via biotin-phalloidin, we observed events with long-distance transportation (>100 µm; Fig. S3) at ionic strengths up to 80 mM in the absence of viscosity enhancing methylcellulose.

**Figure 2:**
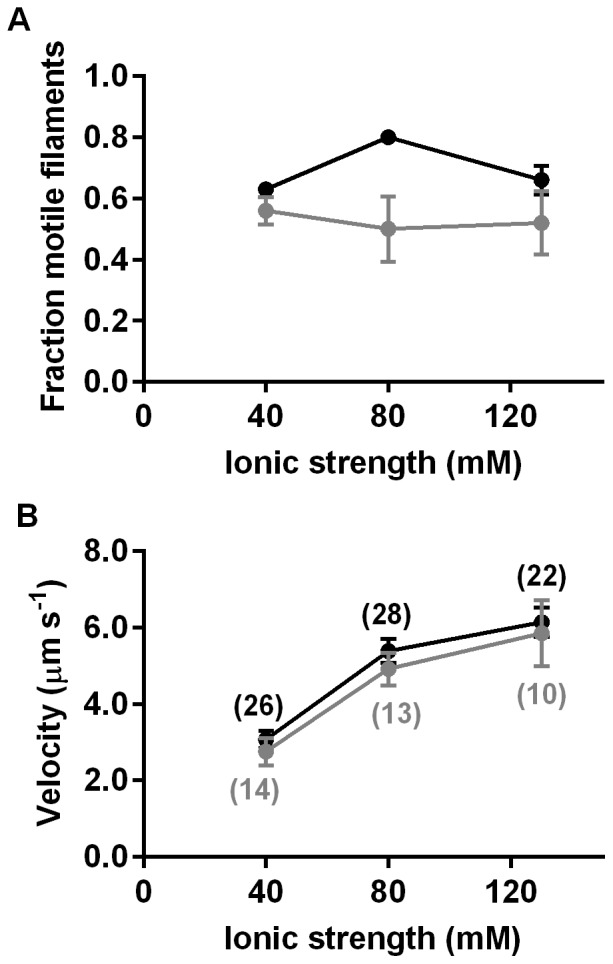
Motility quality for biotinylated actin filaments with 1–2 quantum dot cargoes attached along the filaments. **A.** Fraction of motile filaments in the absence (black) and presence (grey) of quantum dots. Error bars represent SEM. **B.** Sliding velocity for biotinylated F-actin without (black) and with (grey) quantum dots. Error bars represent 95% confidence intervals. Number of filaments given in parentheses.

The effects of quantum dot loading on HMM induced actin filament sliding at varying ionic strengths are illustrated in Fig. 2 for experiments distinct from those in Table 1 (and at higher temperatures than these). In accordance with data in Table 1, the fraction of motile filaments (Fig. 2A) tended to be reduced, whereas the velocity (Fig. 2B) was minimally affected by quantum dot attachment at all ionic strengths tested.

**Figure 3:**
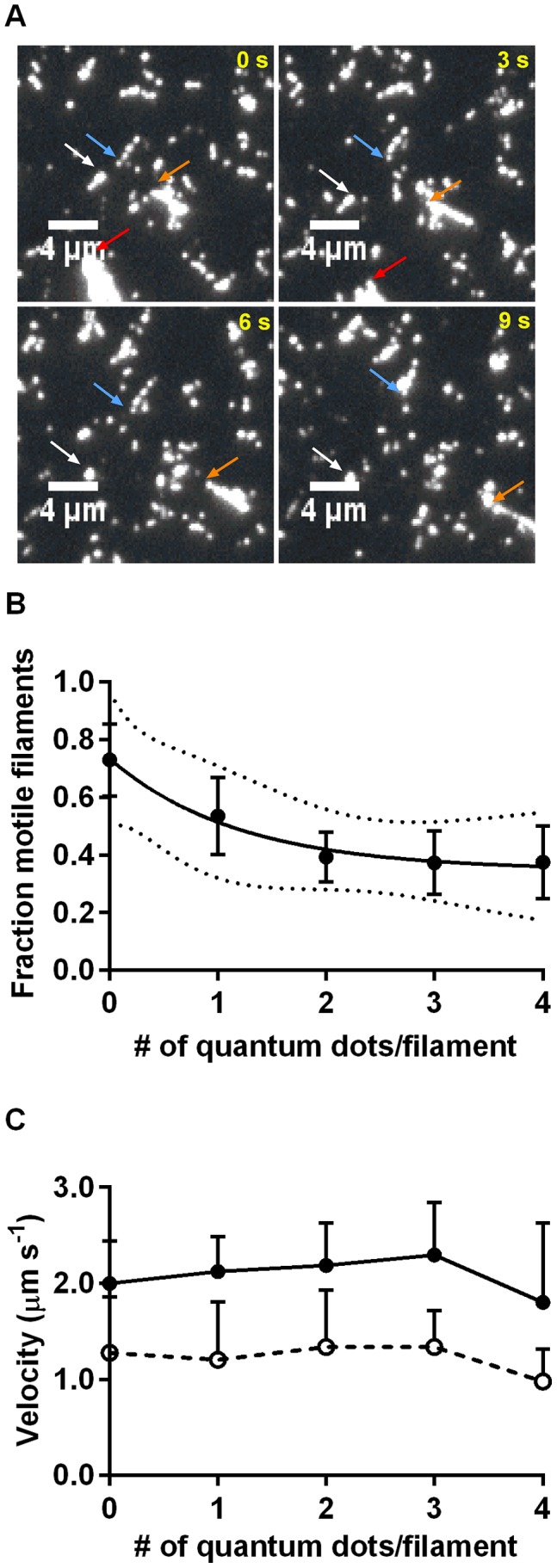
Motility quality vs. number of attached quantum dots on HMM propelled filaments. **A.** Fluorescence micrograph time series at 3 s intervals showing several motile and some non-motile and heavily quantum dot labelled actin filaments. All fluorescence in the micrographs is attributed to the quantum dots. The colour coded arrows point to some motile filaments/aggregates with 5 quantum dots (blue arrow; apparently single filament), >5 quantum dots and presumably 2 cross-linked filaments (orange arrow), small aggregate (white arrow), large aggregate (red arrow). **B.** The average fraction of motile filaments in 3 different experiments. Error bars: SEM (n = 3 different flow cells and experimental occasions for different number of quantum dots; >3–11 quantum dot-labelled filaments per flow cell). The solid line (included for descriptive purpose) represents a single exponential function decaying with increased number of quantum dots to a plateau value. Optimal parameter values and 95% confidence interval (dotted lines) obtained by non-linear regression analysis. **C.** The mean sliding velocity ±95% confidence intervals in 3 (or 4; for 1–3 quantum dots) different experiments. The filled symbols and solid line represent the ten frames running average of the frame-to-frame velocities with lowest CV on the condition that CV<0.5. The open symbols and dashed line, on the other hand, represents average velocities calculated by dividing the integrated sliding distance with the tracking duration (11–14 s; shorter in the few cases when filaments moved out of the image).

In another set of experiments, biotinylated actin filaments were incubated with quantum dots under conditions resulting in 1 to >5 quantum dots per filament and also to the formation of cross-linked filament aggregates (Fig. 3A, Movie S4). It can be seen in Fig. 3B that the fraction of motile filaments decreased with an increased number of quantum dots in the range 1–4, whereas there was negligible effect on sliding velocity (Fig. 3C). This lack of effect on velocity remained during up to 10 min observation period (Fig. S4A).

Motility (sometimes good) was also observed for larger number of quantum dots, but in these instances (cf. Fig. 3A, Movie S4) it was usually difficult to count the exact number of quantum dots or clarify to what degree the filaments were aggregated via quantum dot-streptavidin-biotin bonds. Therefore, no quantitative estimates of motility quality were obtained for >4 quantum dots per filament. Neither did we attempt detailed studies of the concentration dependence of the degree of quantum dot binding to the actin filaments. The short incubation times and rather low quantum dot concentrations used here were chosen to limit the degree of non-specific binding outside the filaments. The limitation on actin binding in this case was not saturation of binding sites but rather the slow diffusion of the comparatively large quantum dots to the binding sites on actin [71].

In some random instances, two parts of a filament (e.g. leading and trailing; Fig. S5) were loaded with several quantum dots, whereas the remaining part(s) of the filament was unlabelled. Filaments of this type moved over large distances without appreciable tendencies for stops and pauses. For instance, the filament in Fig. S5 that initially had 4–5 quantum dots at each end (2 in front end detached after about 10 µm of sliding), moved for >>100 µm at an average velocity of 1.72 µm/s compared to 1.73±0.096 µm/s (n = 18 filaments) in the absence of quantum dots in the same flow cell.

An additional observation of interest was that one filament with two quantum dots executed nearly continuous counter-clockwise rotation for 164 s, apparently with one of the actin-attached quantum dots as an axis. Altogether, 30 full rotations in the same direction were observed. Similar behaviour is observed for one filament in Movie S4 (in the lower right, second half of the movie).

### Liposomes can be Transported at High Sliding Velocity

**Figure 4:**
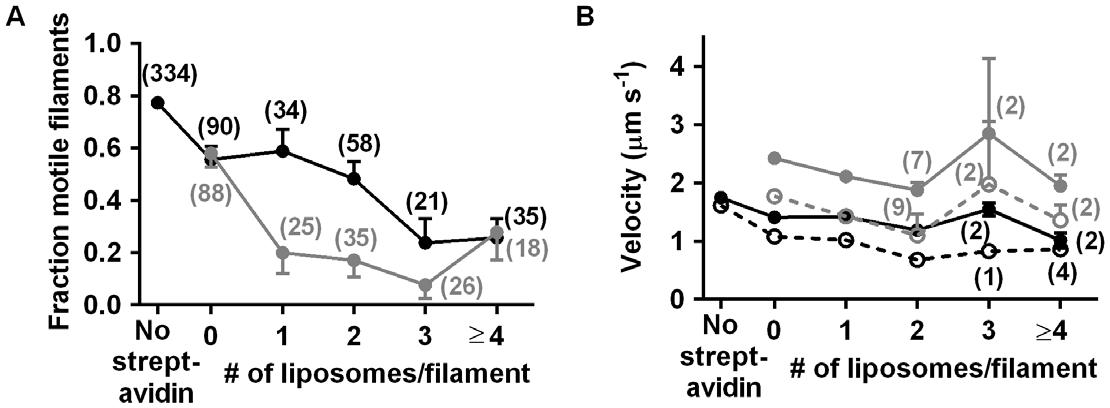
Motility quality vs. number of attached liposomes on HMM propelled filaments. A. The fraction of motile filaments at two different experimental dates (black and grey). **B.** The sliding velocity. Velocity data represented by black and grey represent two different experiments. The filled symbol and solid line represent velocities obtained from the ten frames running average of the frame-to-frame velocities with lowest CV (CV<0.5), while the open symbols and dashed line represents the average velocity as described in Fig. 3. The numbers in parentheses represent the number of filaments studied for each condition (>10 in B unless otherwise stated). Error bars: SEM. Temperature: 21°C.

Streptavidin-mediated attachment of biotinylated liposomes (diameter >∼50 nm) along actin filaments had greater effect on HMM induced sliding than quantum dot attachment. In this case, a reduced fraction of motile filaments (Fig. 4A) with increased number of attached liposomes was associated with slightly reduced velocity (Fig. 4B; Movie S5). Large liposomes (>100 nm in diameter) occasionally caused filament detachment from the surface. The average velocity of filaments with one attached liposome did not change appreciably over a 10 min period as tested in one experiment (Fig. S4B). Most actin filaments in the experiments with liposomes presumably had several more streptavidin molecules attached than those required to link the liposome to the filament, possibly contributing to lower fraction of motile filaments (see above).

In some experiments, we observed large liposomes (as suggested by high fluorescence intensity; Movie S6) that appeared to roll on the surface with a velocity similar to that for filaments without liposomes. The actin filaments in such experiments, observed by switching to a TRITC filter-set, appeared as ellipsoids or spheres with greater width than other filaments in the image (Movie S6). A possible interpretation is that one actin filament was bent around the liposome in a way that the bend and two ends of the filament are not fully resolved in the microscope. Indeed, the actual formation of such a bend was directly observed in one case. Alternatively, in other cases two or more actin filaments may have attached on the liposome. This is similar to a strategy systematically exploited for liposome transportation in a recent study [41].

### CapZ is an Alternative to Gelsolin for Attaching Cargos to the Plus End of Actin Filaments

**Figure 5:**
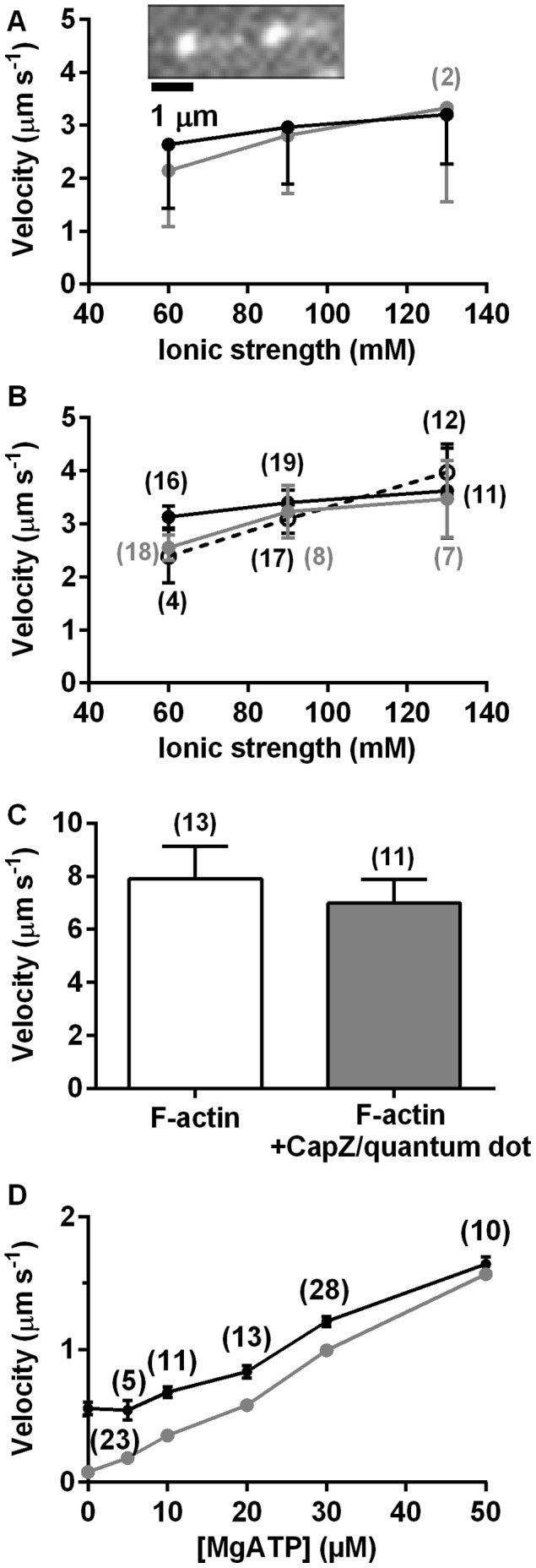
Effect of CapZ binding to F-actin on HMM propelled actin filament velocity. A . Sliding velocities at different ionic strengths for F-actin with (grey) and without (black) CapZ/quantum dot complex (n = 3 experimental occasions and different HMM preparations unless indicated in figure; >17 filaments analysed for each condition). Temperature: 22°C. *Inset*: two subsequent images (interval: 1.6 s) of HMM propelled RhPh-labelled F-actin (sliding to the right) with quantum dot attached to the trailing end via CapZ. **B.** Sliding velocities for F-actin capped with CapZ-rhodamine (dashed line, open symbols), CapZ/quantum dot complex (grey) and without CapZ (black) in one given experiment (number of filaments in parentheses). **C.** Effects of CapZ on sliding velocity at high temperature (28.6°C) in AMc130 assay solution. Average sliding velocities for F-actin in the absence of CapZ or in the precence of CapZ/quantum dot complex. Filament lengths of F-actin without CapZ limited to the mean length of CapZ capped actin filaments ±2 standard deviations (0–3 µm; see further Fig. S6, Fig. S7) to ensure comparability. Error bars in A–C: 95% CI. **D.** Velocity measurements (5 frames s^−1^) at low [MgATP] using nanometer tracking of quantum dots attached to CapZ (grey) or tracking the filament centroid (black) for the same filament (number of filaments in parentheses). Note appreciable overestimation of velocity at low [MgATP] by centroid tracking due to noise [66] as most clearly indicated by the tracking of stationary filaments. The tracking of stationary quantum dots using the Gaussian fit (nanometer tracking) suggests a precision of <5 nm in each position estimate. This is based on the apparent speed of stationary quantum dots of 78 nm/s, a frame rate of 5 s^−1^, the fact that the speed is a scalar quantity in the analysis and that each distance between frames, used for the velocity calculations, depends on two position estimates by Gaussian fits.

Here, we describe the first *in vitro* motility assay study using actin filaments with CapZ attached to the actin filament plus end (according to search on PubMed September 22, 2012). Poly-histidine tagged CapZ was expressed in *E. coli* [54] and purified (Fig. S2), as described previously [54], [55]. For our experiments, CapZ was either biotinylated or labelled with rhodamine, using NHS-ester based conjugation chemistry. Both the α- and β subunits of the CapZ heterodimer were labelled by these procedures as suggested by fluorescence following gel electrophoresis (rhodamine) or estimation of molar mass by SDS-PAGE. This labelling procedure did not inhibit the actin capping capability (Fig. S6) and allowed production of CapZ capped actin filaments by polymerisation in the presence of CapZ. Quantum dots (incubation with 1–10 nM for 30 s - 1 min) were attached to the biotinylated CapZ after immobilization of the capped actin filaments on a HMM coated surface. Considering the effects of CapZ on actin filament length (Fig. S6), it is reasonable to presume that a majority of the filaments are capped with CapZ. The fact that <5% of the filaments bound quantum dots is most likely attributed to slow diffusion during a limited incubation period. This is in accordance with the low quantum dot binding along biotinylated actin filaments (see above). After quantum dot binding, an ATP-containing assay solution was added to observe motility (Movie S7). It can be seen in Fig. 5A–B (from overlapping 95% confidence intervals) that CapZ attachment was without consistent effect on HMM propelled actin filament velocity. This was tested and found to apply within the range of ionic strengths where a noticeable effect of gelsolin was seen in a recent study [49]. For practical reasons these results were obtained at rather low temperature (room temperature ∼ 22°C). However, in a control experiment the effect of CapZ was also studied at higher temperature (∼ 29°C, Fig. 5C). Neither in this case did the presence of CapZ significantly affect the sliding velocity provided that correction was performed for the fact that the filaments with CapZ were shorter. This is important because length *per se,* affects velocity (see [72] and Fig. S7). In the *in vitro* motility assay experiments with CapZ, the presence of the capping protein was either verified by the attachment of streptavidin coated quantum dots to biotinylated CapZ or by covalent rhodamine labelling. The fraction of motile filaments was not appreciably affected by the presence of CapZ and the fraction of motile filaments was higher than 50% for actin filaments that had quantum dots attached via CapZ.

The fact that CapZ did not affect the sliding velocity suggests that it should be useful to mediate attachment of quantum dots or magnetic particles [10] in fundamental studies of actomyosin function. Here, we exemplify this potential usefulness by demonstrating tracking with nanometer accuracy [47] of quantum dots attached to CapZ on HMM propelled actin filaments (Fig. S8). This allowed us to obtain accurate MgATP-velocity information at low MgATP concentration with frame rates as high as 5 s^−1^ (Fig. 5D). Thus, the plot of velocity against [MgATP] is nearly linear in the range of 5–30 µM MgATP and extrapolates to zero as expected for a hyperbolic relationship between velocity and MgATP-concentration [67], [73]. The non-zero velocity at [MgATP] = 0 µM reflects the approximately 5 nm uncertainty in each position estimate used for the velocity calculation. This is, however, considerably less than the uncertainty in the centroid tracking method [66]. As also shown in Fig. 5D, this centroid tracking method gives an apparent velocity of approximately 0.5 µm/s at 0 µM MgATP and also considerably higher velocities at micromolar MgATP concentrations than expected for a hyperbolic relationship.

## Discussion

### Methodological Issues

The fraction of motile filaments, the velocity of smoothly sliding filaments (CV<0.5),) and the average velocity report different aspects of actomyosin function. The “smooth” velocity characterizes the optimal function under the condition studied. The average velocity, on the other hand, reports the velocity that would be most representative for the behaviour of an ensemble of moving filaments, e.g. in a nanodevice. Thus, in this case filaments were not selected based on the smoothness of sliding – all filaments were included even those that were stationary for all or a very small fraction of the measurement period. Importantly, the filaments moved for appreciably longer times and distances than generally used for velocity measurements (Fig. S3 and Fig. S4) and there was no substantial change in motility (velocity and fraction of motile filaments) over the periods (usually around 5 minutes) when measurements were made. This should be compared to less than 1 minute required for actomyosin driven concentration on a nanostructured surface [74]. Additionally, as the velocities did not change with time, measurements over short time periods for a large number of randomly selected filaments would be just as representative for the average velocity of the filament ensemble as measurements over more extended time periods.

### Relation to Earlier Results

Covalent modification (by NHS-biotin) of up to 4 lysines per actin monomer affects neither the capability for actin polymerization nor the actomyosin motor function (see also Kumar et al. [32]). This brings new possibilities for extensive covalent modification of the lysines also for other purposes, e.g. attachment of antibodies at high densities along the actin filament for use in diagnostics applications [7], [31], [32]. The success of covalent biotinylation is also important for certain fundamental studies of actomyosin function [75], [76] because the previously used alternative actin-phalloidin link [11], [60] to couple biotin to actin has a limited half-life of <30 min [77]. The present motility assays with quantum dot attachment to covalently biotinylated actin filaments could, unlike those with biotin-phalloidin mediated quantum dot-attachment [11], be performed in the absence of viscosity enhancing methylcellulose in the assay solution at ionic strengths up to 80 mM (where actomyosin interactions are rather weak) without appreciable detachment of the actin filaments. This considerably improved motility suggests that effective actomyosin driven cargo-transportation is easier to achieve with side-attached cargoes than usually believed (cf. [7]). It is unclear whether the improved motility compared to the previous study [11] is due to geometrical differences between the two modes of biotinylation or to recent changes in commercial preparation of the streptavidin coated quantum dots (not revealed by Invitrogen for proprietary reasons). In spite of the improved motility compared to previous work [11], our results are, nevertheless, consistent with findings [39], [51] that substantial cargo-loading of actin filaments with cargoes having diameters of tens of nanometers would give lower fraction of motile filaments. Importantly, however, we found only small effects on motility with small cargoes (of biotin-streptavidin size), even when the loading was quite extensive. The lower fraction of motile filaments with liposomes than with quantum dots supports the view (cf. [37]) that cargo-size is an important factor. Therefore, with large cargoes, unipolar actin bundles where the actin filaments are cross-linked via fascin [78], may be more effective [15], [37] as HMM propelled shuttles than isolated actin filaments despite the increased complexity imposed by bundle production and need to maintain bundle integrity over time.

The effects of cargoes along actin filaments on actomyosin motility were generally of the all-or-none type. That is, if motility was inhibited it was usually completely switched off, at least temporarily, whereas only limited effects on sliding velocity were seen for motile filaments. This seems to be different from the inhibition of motility of microtubules propelled by conventional kinesin 1, where a substantial reduction in velocity was observed with increasing streptavidin loading of the filament [29], [79]. These differences may be related to the fact that myosin II, in contrast to conventional kinesin motors, is non-processive. The processive motion of kinesin along microtubules would be temporarily blocked at a cargo “road-block” [29], but the kinesin motor could overcome the road-block without detaching [29], translating into a reduction of velocity for an ensemble of kinesins. In contrast, the detachment of a substantial fraction of active myosin II motors could lead to temporary or long-term switching off of motility and even detachment from the surface. The lack of cargo-effects on the sliding velocity might at first seem surprising, particularly in the presence of methylcellulose where drag forces might be of relevance. However, methylcellulose alters the macroscopic viscosity, but not the microscopic viscocity and therefore does not inhibit sliding filament velocity [72], [80]. Also, a simple calculation suggests that the drag forces should be very small in any case. Thus, if a quantum dot is approximated by a sphere, the drag force (F_drag_) is given by the relationship F_drag_ = 6πηrv_f,_where r is the quantum dot radius, v_f_ is the sliding velocity and η is the viscosity (<150 cP for the type and concentration of methylcellulose used here) [72]. Now, inserting r<10 nm and v_f_<10 µm/s it follows that the viscous drag force on a quantum dot is less than 0.3 pN, i.e. considerably lower than the average force per myosin head of several pN [81]. In addition, at the HMM surface densities, ATP concentrations and velocities used here there are on the order of 10 myosin heads attached per µm of the filament at each given time [42]. Under these conditions the drag force would also be negligible for liposomes with radius five times higher than for a quantum dot.

### More on Cargo Transportation with Side-attached Cargoes

In contrast to the lack of effects of covalent biotinylation, an increased fraction of stationary filaments and temporary stops in the sliding was observed upon increasing streptavidin, quantum dot and liposome loading. Only small effects were seen with up to ∼50 streptavidins per µm of the actin filament. However, the effect was noticeably enhanced with increased cargo size and for a given number of cargoes. Thus the fraction of motile filaments progressively decreased from streptavidin over quantum dots to liposomes. This was the case in spite of a streptavidin layer on both the quantum dots and the liposomes. The inhibiting effects of the cargoes may be attributed to different forms of non-specific interactions with HMM [43], [82], [83] and/or the prevention of myosin binding to actin. However, the HMM-quantum dot interactions seem to be weaker than interactions between quantum dots and the underlying TMCS-surface. Thus, increased non-specific surface binding of quantum dots was observed at low HMM incubation concentrations (in spite of the presence of BSA) or when myosin subfragment 1 was used as a motor fragment instead of HMM (unpublished observations). However, extensive interactions between quantum dots attached to actin filaments and the TMCS-surface are unlikely at the high HMM incubation concentrations (50–120 µg/ml) used here. This is attributed to high HMM surface density [42], [43], [84] that would be expected to block access to the surface (cf. [83]). Additionally, quantum dots attached to actin filaments are more than 20 nm away from the surface due to the thickness of the HMM layer. This further limits the probability for direct surface interactions [43]. Finally, interactions with non-fluorescent blocking actin filaments [42] seem to be without relevance because omission of blocking actin did not appreciably modify the quantum dot effects on motility.

Different possibilities to account for the temporary stops and a larger fraction of stationary filaments in the presence of cargoes may be considered. First, bulky cargoes as well as a large number of small cargoes may block access of HMM to binding sites on actin, consistent with a tendency for filaments loaded with large pieces of cargo to temporarily detach from active HMM molecules. This effect may be alleviated by annealing actin filament fragments with and without cargoes to produce continuous cargo-free stretches (cf. [12], [39]) as indicated in Fig. S5. Non-specific interactions between cargoes and the underlying HMM coated surface most likely contribute to motility inhibition. The non-specific interactions may include steric trapping of cargoes in spaces between HMM molecules. The idea of such trapping, without rigid surface attachment of the quantum dots, is consistent with the observation of several sequential rotations in the same direction of non-sliding actin filaments around actin-attached quantum dots (see above and Movie S4). Electrostatic interactions between the negative net charge of streptavidin at pH 7.4 and a positive electrostatic surface potential in the actin binding region of HMM [85], [86] is unlikely to be of appreciable significance for the motility inhibition with few quantum dots because numerous streptavidin molecules were attached to actin filaments with only minimal effects on motility (cf. [29]). However, other types of non-specific interactions, e.g. hydrophobic, between the quantum dot itself and HMM, may be important. Here, it would be of interest to study how the chemical characteristics of the cargoes affects actomyosin function as studied recently for kinesin-microtubule cargo transportation [79].

### Cargo-transportation in Relation to Mechanisms of Motion Generation and Actin Dynamics

A large fraction of filaments with 1–2 quantum dots and liposomes were observed to slide throughout the observation period (often >100 µm). Moreover, even rather large (>100 nm diameter) cargoes and/or several cargoes per filament were occasionally transported with minimal effects on velocity suggesting that rotation around the filament long axis during sliding is not of critical importance for chemomechanical energy transduction (cf. [45] and compare Fig. S1). The maintained high sliding velocity with heavy streptavidin loading of actin filaments is also consistent with this idea. This finding also argues against Brownian motor models [87] that explain actomyosin force generation by biased diffusion of myosin heads between binding sites with progressively lower energy on subsequent monomers along the actin filament. The similar size of streptavidin compared to the actin monomer and streptavidin labelling of almost 20% of the monomers would (assuming uniform distribution of streptavidins along the filament) limit the average number of diffusional steps of a myosin head along the filament to less than 2 before detachment at a streptavidin molecule. This is clearly less than proposed recently for a Brownian motor model [87]. If movement in such a model would occur along a given protofilament, the maximum distance before detachment with 2 steps would be about 10 nm, clearly less than the value of up to 30 nm at low load observed experimentally by Kitamura et al. [88].

For the use in studies of actomyosin function (e.g. [33]) it is important that attachment of e.g. fluorescent tags or other cargoes via plus end capping proteins does not affect actomyosin kinetics. Such effects may be expected for gelsolin mediated attachment if the kinetics is critically dependent on the actin filament structure since this structure is modified by interactions of gelsolin with subdomains 1 and 3 of the actin monomer [50], [51], [52], [53]. Indeed, a study [49] using both Ca- and Mg-actin filaments nucleated in the presence of gelsolin seem to accord with effects of gelsolin on motility, whereas another study [33] did not detect such effects. Therefore, more studies are needed to resolve this issue. Further, the possibility to use fragments of gelsolin [50] or other capping proteins within the gelsolin family [89], [90] may also be considered. Here we found no effects on motility of CapZ binding to the actin filament plus end. This is consistent with minimal structural changes along the actin filament upon CapZ binding, as expected because CapZ, unlike gelsolin, does not sever the filament. The usefulness of CapZ mediated attachment of quantum dots to actin filaments for future detailed functional studies of actomyosin is exemplified in Fig. 5D.

### Cargo-transportation in Diagnostic Nanodevices

For use in diagnostic nanodevices, antibody coated actin filaments have recently [31], [32] been proposed for capturing analyte molecules from solution for subsequent rapid motor driven concentration to a detector site on a nanostructured surface. Our finding that close to 20% of the actin monomers may be loaded with protein-sized cargoes without appreciable effect on motility is therefore essential. However, the use of antibody-coated filaments for capture of analytes would be of significance particularly at low analyte concentration when the need for signal amplification by actomyosin mediated concentration at a detector site is crucial. Under these conditions, very few (e.g. one or two) antibody-analyte complexes of sizes similar to quantum dots would be bound to each actin filament. We have demonstrated here that such limited cargo loading has quite small effects on motility quality.

### Conclusions

We have demonstrated fast actomyosin based cargo transportation under a range of conditions using side-attached and end-attached model cargoes with different sizes similar to those of diagnostically relevant biomarkers and nanoparticles. The results, together with recent demonstration of long-term storage of actomyosin based nanodevices [91] guiding of actomyosin motility along nano-tracks [18] and covalent antibody attachment to actin filaments [31], [32] support the usefulness of actomyosin for nanoseparation in medical diagnostics devices. The results with streptavidin loading also have potential implications for mechanistic models of actomyosin based motion generation and the effect may deserve further investigation. Finally, our results favour the use of CapZ, rather than gelsolin for mediating attachment of e.g. quantum dots or magnetic particles to the trailing end of actin filaments in various fundamental studies.

## Supporting Information

Figure S1 **Schematic illustration of streptavidin, quantum dots and liposome attachment to F-actin used in present work.** **A.** Attachment of quantum dot and liposome along actin filament via biotin-streptavidin links**. B.** Attachment of quantum dot via a plus-end binding protein in the form of biotinylated CapZ. Arrow indicates direction of movement in the in vitro motility assay. Figure approximately to scale.
(TIF)

Figure S2 **SDS-PAGE of CapZ, biotinylated CapZ and actin.** **A.** Novex® 4–20% gradient tris-glycine acrylamide gel (Invitrogen) was run under reducing conditions and stained using Novex® colloidal blue staining kit (Invitrogen). Sample preparation, running conditions and staining procedure were performed according to manufacturer’s protocol. Lane 1– Purified CapZ expressed in *Escherichia coli* (1 µg, two subunits with 39 and 42 kDa apparent molecular weights), Lane 2– biotinylated CapZ (1 µg, 4–6 biotins/CapZ molecule). Lane 3– rabbit skeletal actin (1 µg, 48 kDa apparent molecular weight). Lane 4– Protein standard SeeBlue® Plus 2 (Invitrogen). The apparent molecular weights in kDa are indicated on the gel. Lane 5– purity control for CapZ (10 µg). Lane 6– purity control for biotinylated CapZ (10 µg). Lane 7–purity control for rabbit skeletal actin (10 µg).
(TIF)

Figure S3 **Matlab program output after tracking HMM propelled F-actin with long-distance transportation of quantum dot.** This quantum dot was attached to biotinylated actin filament and the assay solution was a80. **A.** Sliding distance plotted against time during 28.2 s tracking period with a total distance of 120.4 µm. Average velocity, v_avg_, during this period calculated as the ratio v_avg_ = 120.4/28.2 µm/s ≈ 4.27 µm/s. **B**. Frame-to-frame velocity plotted against frame number (at 0.2 s interval). **C.** Filament path in x-y-plane (250×250 pixels; 330 nm^2^/pixel). **D.** The running average of “smoothest” sliding velocity (minimum coefficient of variation; CV) over ten frames plotted against the number of ten frame windows. **E**. The minimum ten-frame CV of the sliding velocity. Note, the mean velocity in D is updated for each reduction in CV in E to select the velocity of smoothest sliding. **F.** Minimum CV and corresponding mean velocity for 10-frame (CV-10; vmean-10) and 4-frame (CV-4; vmean-4) running averages from measurements illustrated in A–E.
(TIF)

Figure S4 **Velocity of actin filaments with cargoes does not change over a 10 min period.** **A.** The velocity of smoothly sliding (CV<0.5) HMM-propelled actin filaments with 1 to 4 quantum dots in one experiment at different times after onset of recording. See inset for meaning of symbols. **B.** The average velocity (see Materials and Methods) of HMM-propelled actin filaments with 1 liposome in one experiment at different times after onset of recording. Each point in A and B refers to a given filament at the time point considered. Full lines and dashed lines represent regression line and 95% confidence intervals, respectively, both obtained in linear regression analysis.
(TIF)

Figure S5 **Actin filament with 4–5 quantum dots at each end, sliding downwards in the image.** The unlabelled centre of the filament is surrounded by two straight yellow lines. The time interval between the two snapshots is 5.2 s.
(TIF)

Figure S6 **Effects of CapZ on actin filament length distribution.** Monomeric actin was polymerized for 3 h in the absence and presence of CapZ/biotinylated CapZ and labelled with APh. Length measurements were done from images for HMM immobilized actin filaments in the absence of ATP.
(TIF)

Figure S7 **Sliding velocity vs. filament length for actin filaments without CapZ.** The arrow represents the range of lengths (0–3 µm) covered by approximately 95% of the filaments with CapZ, measured when these filaments were bound to HMM in rigor (corresponding to mean ±2 standard deviation of the CapZ actin filament lengths). It is likely that the length was slightly reduced upon addition of MgATP due to filament fragmentation caused by motor induced shearing. Thus, the filaments without CapZ, used for velocity measurements, were only those with lengths in the range 0–3 µm in order to ensure reasonable comparability with the velocity of filaments with CapZ/quantum dots complex. The length of the latter filaments was generally not measured for practical reasons. Temperature: 28.6°C. HMM incubation concentration, 120 µg/ml. AMc130 assay solution. Filament lengths measured from intensity data as described by Sundberg et al. [42] to account for errors due to filament motion during exposure time and diffraction limitation for short filaments.
(TIF)

Figure S8 **Tracking of quantum dots with nm accuracy.** *Intensity profiles (bottom) of* stationary quantum dot (bright spots at top left; 40×40 µm^2^) were fitted by two-dimensional Gaussian function for each image frame (frame rate 5 s^−1^). Brownian motion, tracking errors *etc*. (from tracking in the x-y plane for 1.8 s) gave a variation in position of less than 10 nm suggesting that tracking was possible with <10 nm accuracy.
(TIF)

Movie S1 **HMM-propelled actin filaments coated with streptavidin attached to the filaments via biotinylated actin.** Covalently biotinylated actin filaments (5 nM; monomer concentration; 4 biotins/actin) were immobilized on HMM in the flow cell and then incubated with 1 nM TRITC-labelled streptavidin for 1 min. The actin filaments were also labelled with Alexa-488 phalloidin. The movie first illustrates the TRITC-labelled streptavidin using a TRITC filter set followed by observation of the entire filaments using a FITC filter set (approximately 7–13 s into movie) and finally, the TRITC-labelled streptavidin, again. Note that two filaments self-organize into spools. Main Fig. 1A extracted from movie. Frame rate: 5 s^−1^ (real time) and image size: 85×85 µm^2^.
(MOV)

Movie S2 **HMM-propelled actin filaments coated with streptavidin attached to the filaments via biotinylated actin.** Actin filaments (5 nM; monomer concentration; 4 biotins/actin) were immobilized on HMM in the flow cell and then incubated with 20 nM TRITC-labelled streptavidin for 1 min. The concentration of free biotin to block unoccupied biotin-sites of streptavidin was 40 µM. The actin filaments were also labelled with Alexa-488 phalloidin. The movie first illustrates the TRITC-labelled streptavidin using a TRITC filter set followed by observation of the entire filaments using a FITC filter set at the end. Main Fig. 1B extracted from movie. Frame rate: 5 s^−1^ (real time) and image size: 85×85 µm^2^.
(AVI)

Movie S3 **HMM-propelled actin filaments with streptavidin coated quantum dots attached to the filaments via biotinylated actin.** The actin filaments were also labelled with Alexa-488 phalloidin. The movie first illustrates a few frames with the FITC filter set to observe the entire filaments whereas the remaining frames were obtained using the TRITC filter set to observe the quantum dots with emission maximum at 605 nm. Filaments and quantum dots pseudocolored to indicate their different emission maxima. Note, 1. that only a small fraction of the filaments are labelled with quantum dots and 2. that there is a substantial number of non-specifically bound quantum dots, falsely indicating a large number of stationary actin filaments with quantum dots. From series of experiments in Fig. 2 (a80 solution). Frame rate: 5 s^−1^ (real time) and image size: 85×85 µm^2^.
(MOV)

Movie S4 **HMM-propelled actin filaments with streptavidin coated quantum dots attached to the filaments via biotinylated actin.** The filaments are dually labelled and observed using a filter that simultaneously allows observation of Alexa-488 and quantum dots (emission maximum at 605 nm). Note, large number of quantum dots (>5) attached to, presumably, two cross-linked filaments at top left (no free biotin in assay solution). Note further that one filament with two quantum dots (one at very front) that moves to the right, suddenly stops and begins to rotate counter-clockwise for five consecutive turns. This indicates unconstrained rotation around one of the quantum dots as an axis and continuous driving of this rotation by appropriately located HMM molecules. From series of experiments in Fig.3 (a80 solution). Frame rate: 5 s^−1^ (real time). Image size: 46×33 µm^2^.
(MOV)

Movie S5 **HMM-propelled actin filaments with calcein loaded liposomes attached to the filaments via streptavidin and biotinylated actin.** In order to observe the liposomes the movie was recorded using the FITC filter set. Note, that not all stationary liposomes represent stationary filaments due to some non-specific binding of liposomes to the surface. Note further, 1. single “small” liposomes are rather photobleached, 2. one large liposome appears to “roll” over the surface (mid, top). From series of experiments in Fig.4 (A80 solution). Frame rate: 5 s^−1^ (real time) and image size: 85×85 µm^2^.
(MOV)

Movie S6 **HMM-propelled actin filaments with calcein loaded liposomes attached to the filaments via streptavidin and biotinylated actin.** The actin filaments were also labelled with tetramethylrhodamineisothiocyanate (TRITC)-phalloidin. Initially the recording was made using a FITC filter allowing observation of the liposomes. The filter set was then, approximately in the middle of the movie, switched to a TRITC filter set to allow observation of the actin filaments. Note, apparent rolling of liposome over surface. Frame rate: 5 s^−1^ (real time) and image size: 20×25 µm^2^.
(MOV)

Movie S7 **HMM-propelled actin filaments with quantum dots attached to the actin filaments via biotinylated CapZ at the plus-end.** The actin filaments were also labelled with Alexa-488 phalloidin. The movie first illustrates a few frames recorded with the TRITC filter set to observe quantum dots. For a short sequence in the movie the filter set is changed to FITC to observe the entire filaments whereas the remaining frames were obtained using the TRITC filter set to observe the quantum dots again. Note, that only a small fraction of the filaments are labelled with quantum dots and that a large fraction of the stationary quantum dots were non-specifically attached to the surface. HMM incubation concentration 120 µg/ml. From series of experiments in Fig. 5 (A60 solution). Frame rate: 5 s^−1^ (real time) and image size: 85×85 µm^2^.
(AVI)

Abbreviations S1 **List of abbreviations.**
(DOC)
