# Supplementary figures and images for: Transportation of Nanoscale Cargoes by Myosin Propelled Actin Filaments
Source: PLoS One. 2013 Feb 21;8(2):e55931. doi: 10.1371/journal.pone.0055931 (PMC3578877; doi:10.1371/journal.pone.0055931)

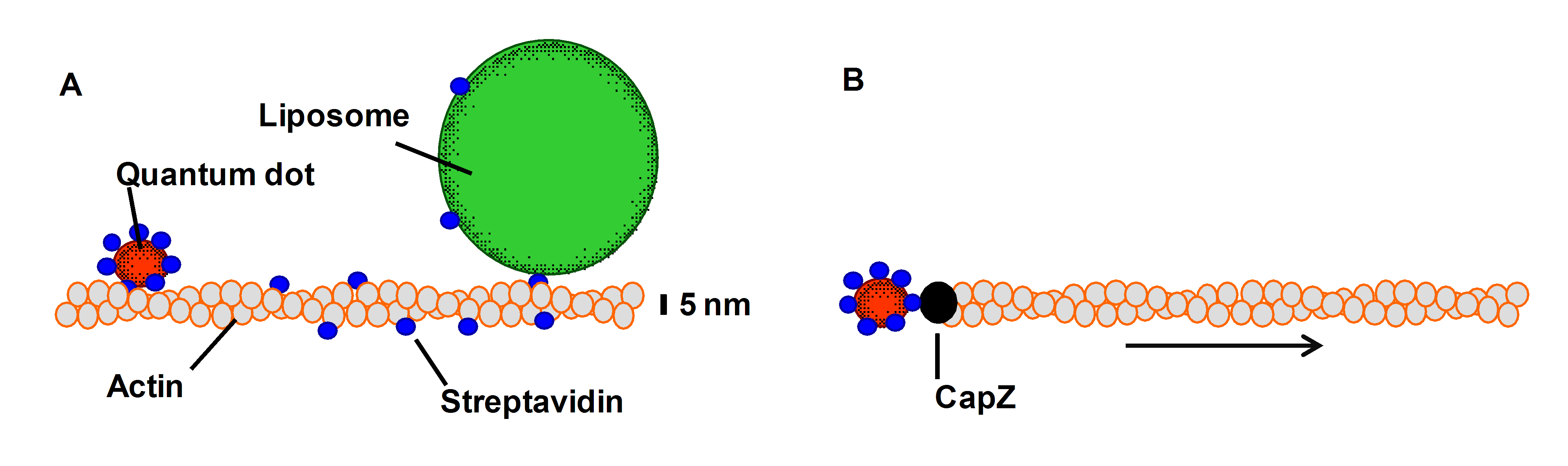

Supplement: Figure S1 — Schematic illustration of streptavidin, quantum dots and liposome attachment to F-actin used in present work. A. Attachment of quantum dot and liposome along actin filament via biotin-streptavidin links. B. Attachment of quantum dot via a plus-end binding protein in the form of biotinylated CapZ. Arrow indicates direction of movement in the in vitro motility assay. Figure approximately to scale. (TIF) [file pone.0055931.s001.tif]

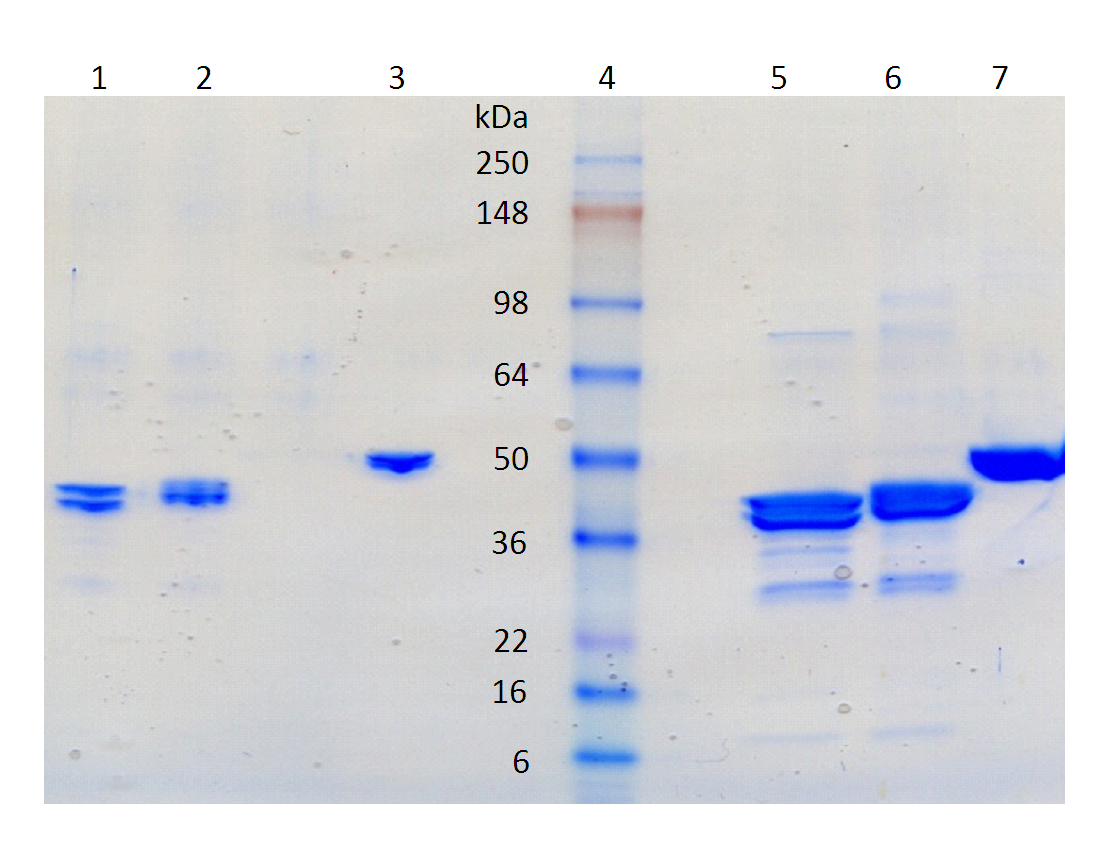

Supplement: Figure S2 — SDS-PAGE of CapZ, biotinylated CapZ and actin. A. Novex® 4–20% gradient tris-glycine acrylamide gel (Invitrogen) was run under reducing conditions and stained using Novex® colloidal blue staining kit (Invitrogen). Sample preparation, running conditions and staining procedure were performed according to manufacturer’s protocol. Lane 1– Purified CapZ expressed in Escherichia coli (1 µg, two subunits with 39 and 42 kDa apparent molecular weights), Lane 2– biotinylated CapZ (1 µg, 4–6 biotins/CapZ molecule). Lane 3– rabbit skeletal actin (1 µg, 48 kDa apparent molecular weight). Lane 4– Protein standard SeeBlue® Plus 2 (Invitrogen). The apparent molecular weights in kDa are indicated on the gel. Lane 5– purity control for CapZ (10 µg). Lane 6– purity control for biotinylated CapZ (10 µg). Lane 7–purity control for rabbit skeletal actin (10 µg). (TIF) [file pone.0055931.s002.tif]

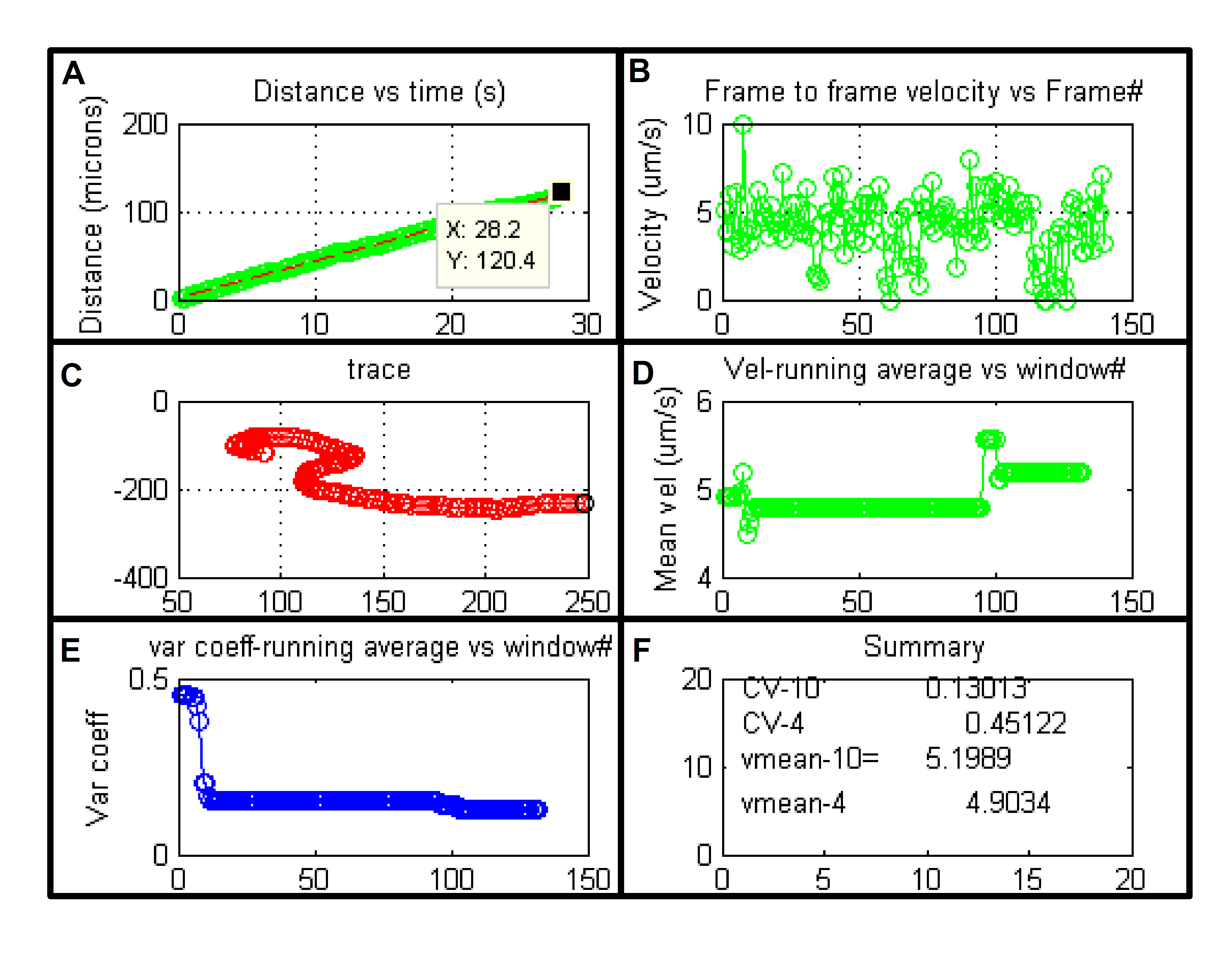

Supplement: Figure S3 — Matlab program output after tracking HMM propelled F-actin with long-distance transportation of quantum dot. This quantum dot was attached to biotinylated actin filament and the assay solution was a80. A. Sliding distance plotted against time during 28.2 s tracking period with a total distance of 120.4 µm. Average velocity, vavg, during this period calculated as the ratio vavg = 120.4/28.2 µm/s ≈ 4.27 µm/s. B. Frame-to-frame velocity plotted against frame number (at 0.2 s interval). C. Filament path in x-y-plane (250×250 pixels; 330 nm2/pixel). D. The running average of “smoothest” sliding velocity (minimum coefficient of variation; CV) over ten frames plotted against the number of ten frame windows. E. The minimum ten-frame CV of the sliding velocity. Note, the mean velocity in D is updated for each reduction in CV in E to select the velocity of smoothest sliding. F. Minimum CV and corresponding mean velocity for 10-frame (CV-10; vmean-10) and 4-frame (CV-4; vmean-4) running averages from measurements illustrated in A–E. (TIF) [file pone.0055931.s003.tif]

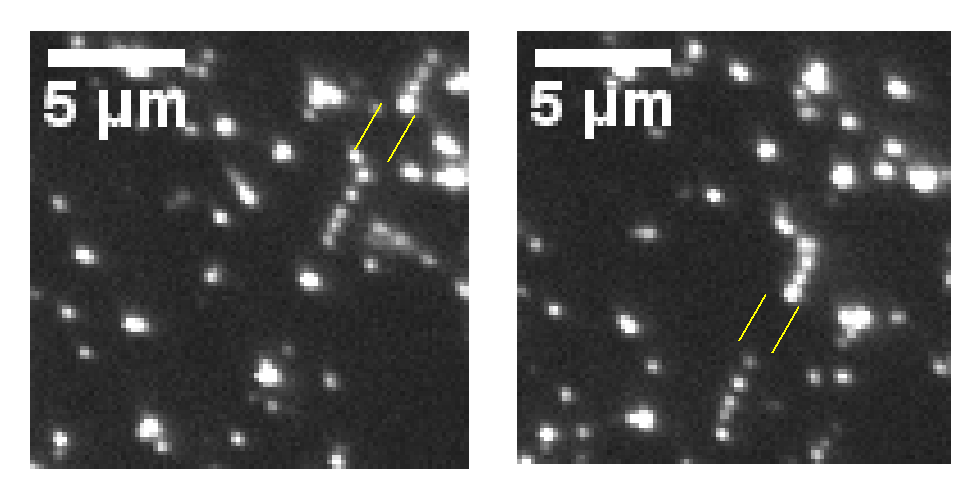

Supplement: Figure S5 — Actin filament with 4–5 quantum dots at each end, sliding downwards in the image. The unlabelled centre of the filament is surrounded by two straight yellow lines. The time interval between the two snapshots is 5.2 s. (TIF) [file pone.0055931.s005.tif]

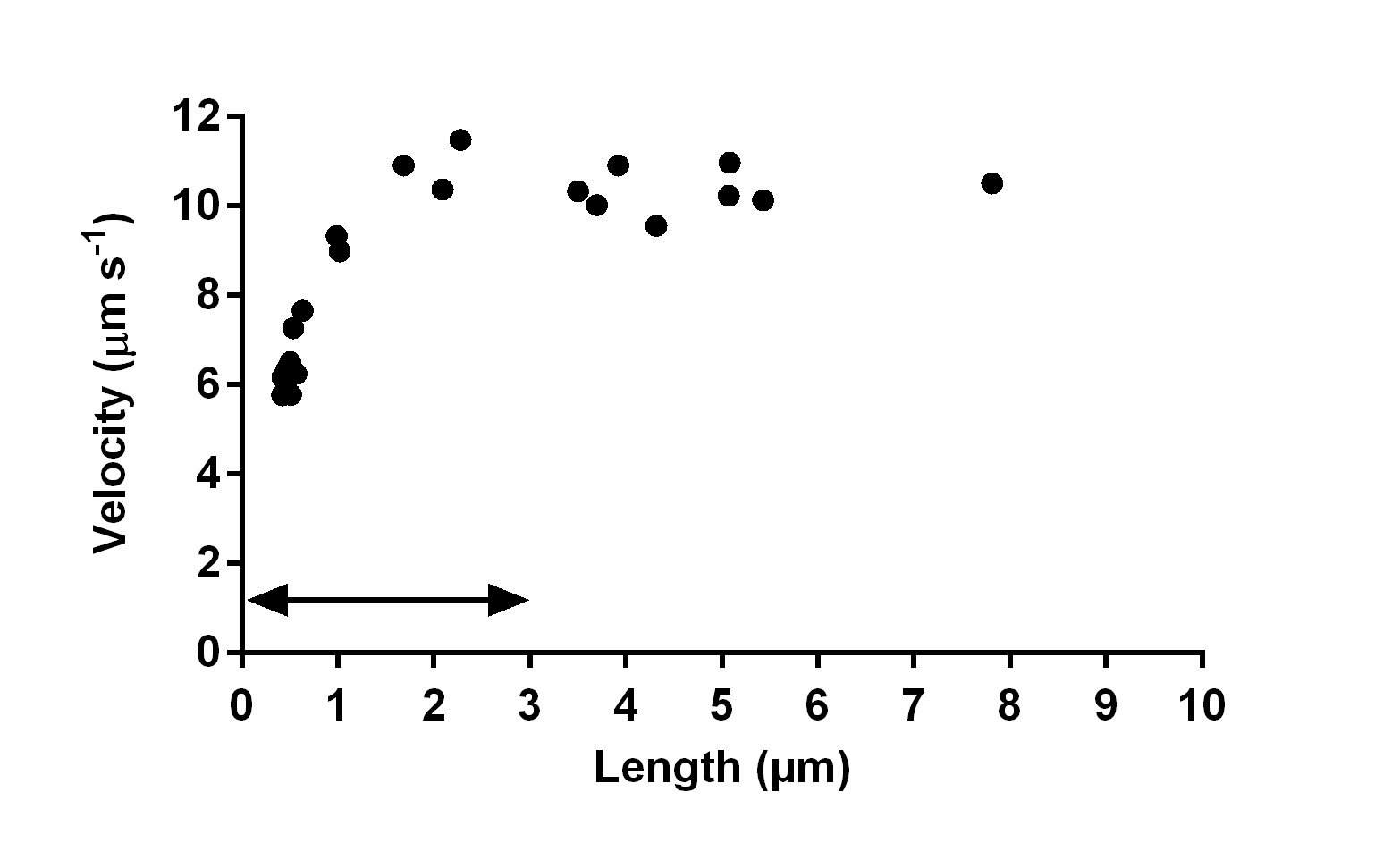

Supplement: Figure S7 — Sliding velocity vs. filament length for actin filaments without CapZ. The arrow represents the range of lengths (0–3 µm) covered by approximately 95% of the filaments with CapZ, measured when these filaments were bound to HMM in rigor (corresponding to mean ±2 standard deviation of the CapZ actin filament lengths). It is likely that the length was slightly reduced upon addition of MgATP due to filament fragmentation caused by motor induced shearing. Thus, the filaments without CapZ, used for velocity measurements, were only those with lengths in the range 0–3 µm in order to ensure reasonable comparability with the velocity of filaments with CapZ/quantum dots complex. The length of the latter filaments was generally not measured for practical reasons. Temperature: 28.6°C. HMM incubation concentration, 120 µg/ml. AMc130 assay solution. Filament lengths measured from intensity data as described by Sundberg et al. [42] to account for errors due to filament motion during exposure time and diffraction limitation for short filaments. (TIF) [file pone.0055931.s007.tif]

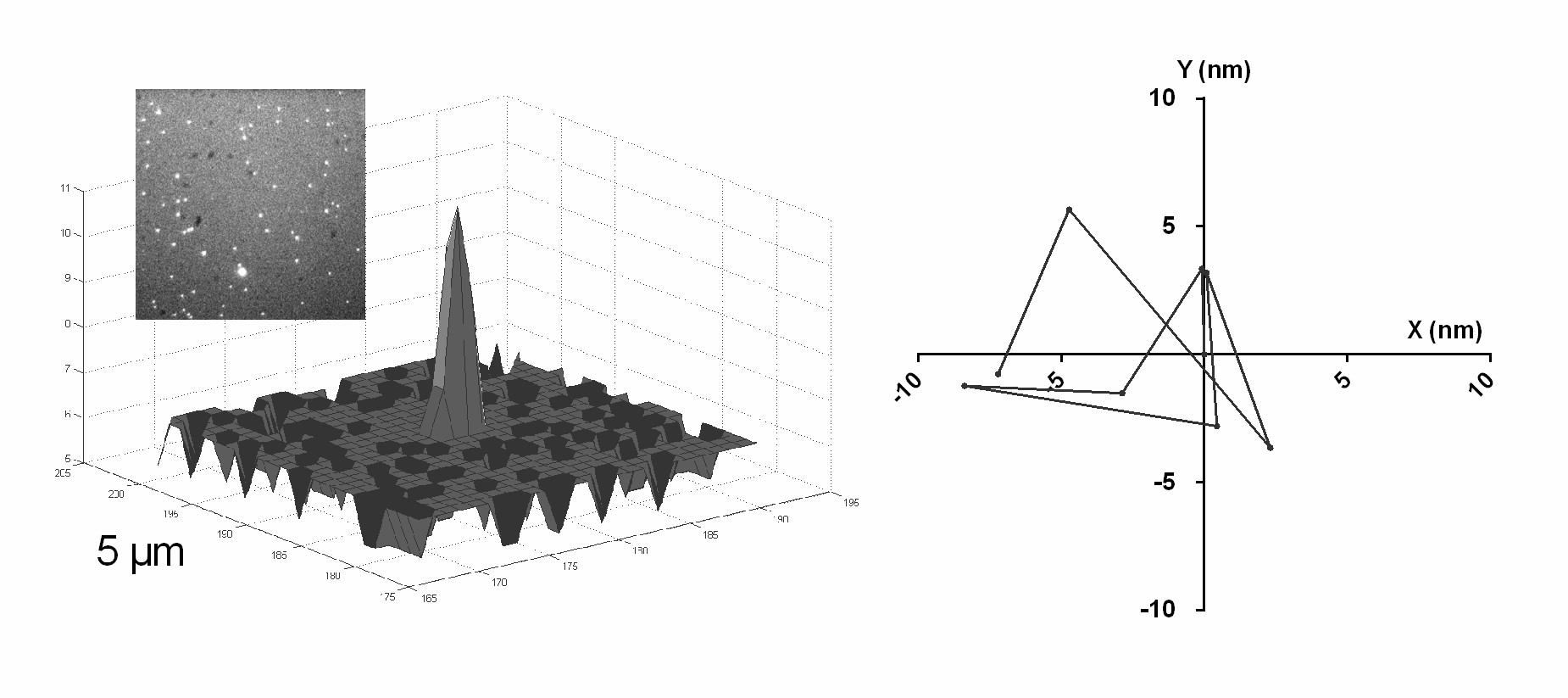

Supplement: Figure S8 — Tracking of quantum dots with nm accuracy. Intensity profiles (bottom) of stationary quantum dot (bright spots at top left; 40×40 µm2) were fitted by two-dimensional Gaussian function for each image frame (frame rate 5 s−1). Brownian motion, tracking errors etc. (from tracking in the x-y plane for 1.8 s) gave a variation in position of less than 10 nm suggesting that tracking was possible with <10 nm accuracy. (TIF) [file pone.0055931.s008.tif]
