# Supplementary material for: Transportation of Nanoscale Cargoes by Myosin Propelled Actin Filaments
Source: PLoS One. 2013 Feb 21;8(2):e55931. doi: 10.1371/journal.pone.0055931 (PMC3578877; doi:10.1371/journal.pone.0055931)
Supplement: Abbreviations S1 — List of abbreviations. (DOC) [file pone.0055931.s016.doc]

**Abbreviations S1.** **List of Abbreviations**

A40 – Assay solution with ionic strength 40 mM

A60 – Assay solution with ionic strength 60 mM

A80 – Assay solution with ionic strength 80 mM

AMc90 – Assay solution with ionic strength 90 mM and 0.5 - 0.6 % methylcellulose

AMc130 – Assay solution with ionic strength 130 mM and 0.5 - 0.6 % methylcellulose

APh – Alexa Fluor® 488 phalloidin

ATP – Adenosine-5'-triphosphate

BSA – Bovine serum albumin (fraction V)

CI – Confidence interval

CV – Coefficient of variation

DTT – Dithiothreitol

*E. coli* – *Escherichia coli*

EMCCD – Electron multiplying charge coupled device

F-actin – Actin filament

FITC – Fluorescein isothiocyanate

G-actin – Monomeric actin

HMM – Heavy meromyosin

NHS – *N*-Hydroxysuccinimide

RhPh – Rhodamine phalloidin

SDS-PAGE – Sodium dodecyl sulphate-polyacrylamide gel electrophoresis

SEM – Standard error of mean

TIRF – Total internal reflection fluorescence

TMCS – Trimethylchlorosilane

TRITC – Tetramethylrhodamine isothiocyanate

Quantum dot – Qdot® 605 Streptavidin conjugate
